# A plasma protein signature for cerebral amyloid angiopathy

**DOI:** 10.1007/s00401-026-03030-5

**Published:** 2026-05-20

**Authors:** Alpana Singh, Marisa N. Denkinger, Antoine Leuzy, Kari Dieckhoff, Jame Liu, Taina M. Marques, Edwin Monuki, Craig Stark, Joshua D. Grill, Christy Hom, David Sultzer, Eric Doran, Ira Lott, Kevin Wood, Brianna Gawronski, Lourdes Gonzalez, Parichita Choudhury, Alireza Atri, Thomas G. Beach, Geidy E. Serrano, S. Ahmad Sajjadi, Kendall Van Keuren-Jensen, Eric M. Reiman, Elizabeth Head, Nicholas J. Ashton

**Affiliations:** 1 Fluid Biomarker Program, Banner Sun Health Research Institute, 10515 West Santa Fe Drive, Sun City, AZ 85351 USA; 2 Department of Pathology and Laboratory Medicine, University of California, Irvine, CA 92697 USA; 3 Institute for Memory Impairments and Neurological Disorders, University of California, Irvine, CA 92697 USA; 4 Department of Neurobiology and Behavior, University of California, Irvine, CA 92697 USA; 5 Department of Psychiatry and Human Behavior, University of California, Irvine, CA 92697 USA; 6 Department of Pediatrics, University of California, Irvine, CA 92697 USA; 7 Department of Neurology, University of California, Irvine, CA 92697 USA; 8 Neurogenomics Division, TGEN, Phoenix, AZ 85004 USA; 9 Banner Alzheimer’s Institute, Phoenix, AZ 85006 USA; 10 Department of Neurology, Mass General Brigham and Harvard Medical, Boston, 92114 USA; 11 Department of Psychiatry and Neurochemistry, Institute of Neuroscience and Physiology, The Sahlgrenska Academy at the University of Gothenburg, Mölndal, Sweden

**Keywords:** Cerebral amyloid angiopathy, ARIA, Blood biomarkers, Neuropathology

## Abstract

Cerebral amyloid angiopathy (CAA) is a cerebrovascular disorder characterized by the deposition of amyloid-β (Aβ) in the walls of leptomeningeal and cortical blood vessels that increases risk of intracerebral hemorrhages and progressive cognitive decline. More than 90% of individuals with Alzheimer’s disease (AD) exhibit some level of CAA. Notably, in the new era of disease-modifying treatments for AD, CAA is a significant risk factor for amyloid-related imaging abnormalities (ARIA), an adverse event associated with anti-amyloid treatments. Therefore, there is great need for accessible, reliable and accurate in vivo biomarkers (e.g., blood-based) to improve antemortem identification of CAA that would improve risk stratification and reduce symptomatic ARIA. In this study, we employed the Nucleic Acid-Linked Immuno-Sandwich Assay (NULISA™) central nervous system panel for exploratory biomarker quantification in antemortem plasma of participants with neuropathological assessments for CAA from the Banner Sun Health Research Institute Brain and Body Donation Program (*N* = 251) and independently validated in the University of California Irvine Alzheimer Disease Research Center cohort (*N* = 110). We evaluated the differential protein expression in antemortem plasma sample taken < 5 years (mean 1.76 ± 1.3) from death using a logistic regression model. We further compared multi-biomarker models and found that a combination of CRP, IL4, CCL11, NPY and PDLIM5, plus demographic covariates showed an area under the curve (AUC) of 0.90 (95% CI 0.86–0.94) to identify neuropathologically confirmed CAA in the discovery cohort. In our independent replication, the antemortem plasma signature performed better than the basic demographics model showing a potential to predict CAA. The exploration and validation in antemortem plasma indicate that a multi-analyte panel, when combined with in vivo blood biomarkers for AD pathology, may be capable of identifying the presence of CAA and could have an meaningful impact on the clinical evaluation of patients under the investigation for cognitive decline. Further developments in biomarkers for this condition are crucial so that CAA identification could inform treatment decisions by highlighting ARIA risk.

**Supplementary Information:**

The online version contains supplementary material available at 10.1007/s00401-026-03030-5.

## Background

The leading cause of neurocognitive disorders is Alzheimer’s disease (AD), which is projected to affect 152.8 million people worldwide by 2050 [30]. AD is characterized by the accumulation of extracellular amyloid beta (Aβ) plaques and intracellular neurofibrillary tangles (NFT), composed of hyperphosphorylated tau (p-tau) [44]. However, in AD, it is uncommon for only Aβ plaques and NFTs to be present as the sole pathological findings at post-mortem. Neuropathological comorbidities are highly prevalent even in early-onset cases, with “pure” AD found in only 44% of individuals under 60 [7], and even more frequent in 8th and 9th decades of life. While Lewy body disease (LBD) is a common comorbidity in younger individuals, the prevalence of other comorbidities, e.g., TDP-43, increases with age. These common comorbidities include cerebral amyloid angiopathy (CAA), a cerebrovascular disorder characterized by the deposition of Aβ in the walls of leptomeningeal and cortical blood vessels that increases risk of microbleeds and intracerebral hemorrhages, and contributes to progressive cognitive decline [3, 18, 32, 45, 70]. CAA is observed in ~ 90% of individuals with AD albeit at differing levels of severity. CAA can also occur independently of AD, and is present in approximately 60–80% of individuals over the age of 80 [8, 50, 59]. The most common form of CAA is sporadic, although rare hereditary forms also exist, typically caused by mutations in the amyloid precursor protein (APP) gene [37, 41, 48, 61].

Recognizing the presence of CAA has long been important in the context of AD, but it has become even more critical with the advent of anti-amyloid therapies for early-stage disease. [27, 54, 69, 81]. These treatments, which target Aβ species in the brain, are associated with amyloid-related imaging abnormalities (ARIA), which can be of the edema (ARIA-E) or hemorrhage (ARIA-H) subtypes. CAA has emerged as a key contributor to ARIA risk, especially in treated patients [17, 71]. In patients undergoing anti-amyloid treatment in clinical trials, the risk of ARIA-E varied from 12 to 40% and ARIA-H was evident in ~ 14% of the participants [27, 69, 82]. While direct evidence linking CAA to an increased risk of ARIA in patients treated with anti-amyloid therapies is limited due to the absence of reliable in vivo biomarkers, the close mechanistic overlap between ARIA and CAA strongly supports this association. Furthermore, ARIA-H and ARIA-E closely resemble CAA and CAA-ri (CAA-related inflammation) on MRI [5, 63].

Therefore, there is great need to identify CAA in vivo not only to aid the clinical evaluation but in also in the potential treatment management to mitigate ARIA risk in early AD. Currently, the definitive diagnosis of CAA is only possible with post-mortem neuropathological assessment [18]. Several neuroimaging biomarkers have been developed for diagnosing and tracking CAA, such as using small vessel disease (SVD) score, cortical superficial siderosis (cSS), and Aβ positron emission tomography (PET) measures [19, 20, 42, 62, 65, 66, 74]. However, neuroimaging biomarkers have an accuracy ranging between 70 to 80% in the updated Boston criteria for CAA [18]. Current clinical criteria for Probable and Possible CAA include MRI identification of heme products and other lesions in specific brain areas via an appropriate sequences but the accuracy of the clinical classification of CAA is limited as sensitivity and specificity are impacted by multiple factors logistical factors in clinical routine [72]. Since neuroimaging biomarkers detect the cerebral hemorrhage, which happens at the final stages of the disease [41, 46], there is an unmet need for less burdensome and more cost effective biomarkers reflecting CAA pathology at earlier stages.

Previous work has explored the viability of Aβ peptides as potential cerebrospinal fluid (CSF) and plasma biomarkers for CAA. Several of these studies observed that CSF Aβ40 is significantly lower in CAA patients compared to AD and healthy older adults, postulating CSF Aβ40 levels as a specific marker of CAA [4, 31, 60, 67, 75]. Contrary to CSF, plasma levels of Aβ40 and Aβ42 perform inconsistently in diagnosing CAA positive individuals [12, 22, 25], likely due to the robustness issues observed for plasma Aβ in detecting brain amyloid plaques and sources of peripheral contamination [9]. In addition to the inconsistent diagnostic and discriminatory accuracy of Aβ peptide levels, existing studies have been limited to genetic CAA cases and, therefore, smaller cohort sizes. Therefore, it is essential to identify novel blood-based biomarkers that can accurately detect and assess the severity of CAA, enabling more accurate diagnosis and potentially minimizing the risk of adverse effects.

In this study, we leveraged antemortem plasma from two well-characterized post-mortem cohorts to evaluate the performance of the next-generation Nucleic Acid-Linked Immuno-Sandwich Assay (NULISA™, Alamar Biosciences) central nervous system (CNS) panel to identify markers of CAA. The Banner Sun Health Research Institute Brain and Body Donation Program (BBDP) served as the *discovery cohort*, including post-mortem-confirmed cases of CAA and antemortem plasma collected within 5 years from death. To validate the discriminative accuracy of these novel biomarkers, we next analyzed an independent *validation cohort* consisting of antemortem plasma samples, also collected within 5 years prior to death, from participants at the University of California, Irvine.

## Materials and methods

### Discovery cohort: brain and body donation program and participant selection

The cases included in this study were contributed by participants in the Arizona Study of Aging and Neurodegenerative Disorders (AZSAND) and Brain and Body Donation Program (BBDP; www.brainandbodydonationprogram.org), a longitudinal clinicopathological research initiative focused on healthy aging, cognition, and movement disorders in the elderly, ongoing since 1997 in Sun City, Arizona [6]. All participants, or their legally authorized representatives, signed an informed consent under an IRB-approved protocol (WCG IRB, Puyallup, WA, USA). We included cases (*n* = 251) with available CAA score and antemortem plasma samples available within 5 years prior to death, without any other exclusions.

### Neuropathological diagnosis of BBDP cohort

A comprehensive neuropathological examination was conducted following standard AZSAND protocols [6]. Histological preparations included large-format (3 × 5 cm), 40–80 µm-thick, cryoprotected frozen sections. Senile plaques, neurofibrillary changes, CAA and other neuronal and glial tauopathies were evaluated using thioflavin S [14]. Neuritic plaque (NP), CAA, and NFT densities were graded blindly as recommended by CERAD with separate semi-quantitative density estimates of none, sparse, moderate, or frequent [52]. All scores were converted to a 0–3 scale for statistical purposes. Regions scored for NP and NFT included cortical gray matter from frontal (F), temporal (T), parietal (P), hippocampal CA1 (H), and entorhinal (E) regions. CAA was scored (0–3 scale) in frontal, temporal, parietal, and occipital lobes of the brain as density scores for amyloidotic blood vessels in standard brain regions. The occipital region was an average of primary visual and association cortex. Large vessel CAA and capillary CAA were considered together. Total CAA score was the arithmetic sum for amyloidotic vessel density of the four cerebral cortex regions. To harmonize the CAA scoring with National Alzheimer Coordinating Center NP forms, the continuous CAA score was stratified into none (0), mild (1–4), moderate (5–8), and severe (9–12). These CAA groups were then analyzed together or separately to assess the association between biomarkers and CAA severity.

### Validation cohort: University of California Irvine

The samples from the University of Califiornia Irvine (UCI) were from participants in the Alzheimer Disease Research Center (ADRC). All participants, or their legally authorized representatives, signed an informed consent under an IRB-approved protocol (2014–1526). Selection of participants to include in the validation study was based on CAA score availability from post-mortem neuropathology assessment and antemortem plasma samples available within 5 years prior to death, without any other exclusions. Neuropathologic examination was performed blinded to clinical information in accordance with current NIA-AA guidelines [40, 53]. Sections were stained for H&E, Aβ (6E10), p-tau (PHF-1), alpha-synuclein and TDP-43. Final neuropathology data are collected using the National Alzheimer Coordinating Center NP forms (NACC forms) [10] where CAA scores (none, mild, moderate, severe) were based upon published criteria [55, 78]. The UCI cohort used ordinal staging systems for amyloid (0, A, B, C) [73] and tau (0, I–VI) [13]. For consistency in the validation analysis, we converted amyloid and tau stages into continuous scales ranging from 0–3 (0 = 0; 1 = A/I–II; 2 = B/III–IV; 3 = C/V–VI). AD status was then defined as the sum of these two scaled variables (range: 0–6). A total of 148 participants aged between 42 to 112 years old were included. As the UCI ADRC also has a Down syndrome (DS) Core, 16 of these samples were from people with DS ranging in age from 42 to 72 years old. Supplementary Table 2 shows the demographics of the UCI ADRC samples included in the study.

### Plasma collection and processing

Plasma samples were collected less than 5 years prior to death. In the BBDP cohort, antemortem plasma samples were acquired in 10 ml K2-EDTA treated BD vacutainers, centrifuged at 3500*g* for 10 min at room temperature within 1 h of the blood draw, aliquoted 500 μl in Certified RNase-, DNase-, and pyrogen-free, polypropylene tubes, and frozen immediately at − 80 °C. In the UCI cohort, samples were acquired in 10 ml K2-EDTA treated BD vacutainers. Samples were centrifuged at 2000×*g*, at 4 °C, for 10 min within 1–2 h of acquisition. Plasma was aliquoted into 500 μl samples in sterile 1 ml medical grade polypropylene tubes and stored at − 80 °C.

### Nucleic Acid-Linked Immuno-Sandwich Assay (NULISA™) analysis

NULISA™ CNS assays were performed as described previously [29]. Plasma samples stored at − 80 °C were thawed on ice and centrifuged at 10,000*g* for 10 min. Exactly 25 μL supernatant was plated in 96-well plates. Briefly, immunocomplexes were formed with DNA-barcoded capture and detection antibodies, followed by a capturing and washing step of the immunocomplexes on paramagnetic oligo-dT beads. The immunocomplexes were then released into a low-salt buffer, which were captured and washed on streptavidin beads. Finally, the proximal ends of the DNA strands on each immunocomplex were ligated to generate a DNA reporter molecule containing both target-specific and sample-specific barcodes. DNA reporter molecules were pooled and amplified by PCR, purified and sequenced on Illumina NextSeq 2000.

### Data processing and normalization

For NULISA, sequencing data were processed using the NULISAseq algorithm (Alamar Biosciences). The sample- (SMI) and target-specific (TMI) barcodes were quantified, and up to two mismatching bases or one indel and one mismatch were allowed. Intraplate normalization was performed by dividing the target counts for each sample well by that well’s internal control counts. Interplate normalization was then performed using interplate control (IPC) normalization, wherein counts were divided by target-specific medians of the three IPC wells on that plate. Data were then rescaled, add 1 and log2-transformed to obtain NULISA Protein Quantification (NPQ) units for downstream statistical analysis. APOE4 was removed from the NPQ file for downstream analysis as it could not be used as a continuous variable, as suggested by the platform developers.

### Statistical analysis

Demographics were summarized descriptively. For the discovery BBDP cohort, the composite CAA score (0–12) was binarized: “Yes” if the composite score was > 0. We modeled log₂-transformed protein levels for all 126 candidate proteins using logistic regression with CAA status as the outcome for the primary analysis, adjusting for age, sex, APOE ε4 carriership, and an Alzheimer’s neuropathology composite (amyloid plaque score 0–15 plus tau-tangle score 0–15, treated as continuous variables). The coefficient from these models represents an adjusted log₂ fold-change. *P *values were adjusted via the Benjamini–Hochberg method, classifying proteins as FDR-significant, nominally significant (*p* < 0.05 but FDR ≥ 0.05), or not significant. To prioritize biomarkers for multivariable modeling, we fit adjusted single-protein logistic regression models with CAA as the outcome on each biomarker plus the same covariates and ranked models by the corrected Akaike Information Criterion (AICc), retaining the five biomarkers with the lowest AICc, and area under the curve (AUCs) with 95% confidence intervals were computed from model-based predicted probabilities. We then compared three nested logistic models: Model 1/Basic model (age, sex, APOE ε4), Model 2/Base model (Model 1 + Alzheimer’s neuropathology composite), and Model 3/Augmented model (Model 2 + the five selected biomarkers). ROC curves were generated consistently across models from empirical sensitivities and specificities based on model-predicted values, without smoothing. Discrimination was assessed with AUC, and we performed DeLong’s test for comparing AUCs. For clinical interpretability, we identified the Youden-optimal probability threshold for each model and reported positive and negative predictive values at those thresholds; beeswarm plots of predicted probabilities by observed CAA status illustrate probability spread. Tests were two-sided with *α* = 0.05, and 95% confidence intervals were presented when applicable. To validate these findings in an independent neuropathology cohort from UCI, we computed the CAA discriminatory accuracy of the five selected biomarkers from the BBDP cohort by comparing the AUCs via ROC analysis: Model 1 (age, sex, APOE ε4), Model 2 (Model 1 + Alzheimer’s status), and Model 3 (Model 2 + the five selected biomarkers). Significant differences among the AUCs were tested measured using DeLong’s test. All analyses were performed in R (v4.1.2).

## Results

### Participant characteristics

The BBDP neuropathology cohort included 251 participants, and antemortem blood collected < 5 years prior (mean [SD], 1.77 [1.26] years) to death with mean [SD] age, 85 [8.20] years, 150 male [60%], and 101 females [40%] (Table 1). In total, 146 participants (58.2%) were neuropathologically confirmed as CAA positive while 105 participants (41.8%) were categorized as non-CAA. Further, 67 participants were *APOE* ε4 carriers while 184 were non-carriers. In total, at the time of plasma sampling, 89 participants were cognitively unimpaired, while the remaining were cognitively impaired. Postmortem neuropathological diagnosis indicated the presence of 152 AD cases (60.56%), 64 TDP-43 cases (25.49%), and 80 Stage III/IV Lewy Body-positive cases (31.87%), alone or in combination with the 146 CAA positive cases. The validation cohort from UCI had 148 participants and antemortem blood collected < 5 years prior to death (mean [SD], 3.78 [0.76] years). The mean [SD] age was 78.92 [13.71] years with 83 males [56.08%], 65 females [43.92%] (Table 1). In total, 79 participants (53.38%) were neuropathologically confirmed as CAA positive (mild, moderate, and severe) while 69 participants (46.62%) were categorized as non-CAA. Further, 59 participants were *APOE* ε4 carriers while 89 were non-carriers. Postmortem neuropathological diagnosis indicated the presence of 84 AD cases (56.76%), 11 TDP-43 cases (7.43%), and 16 Lewy Body-positive cases (10.81%), alone or in combination with the 79 CAA positive cases. Of note, the ADRC sample set also included 18 Down syndrome (DS) individuals, which averaged 58.4 years in age, with 10 males and 8 females and 3 *APOE* ε4 carriers. All of these cases were moderate to severe CAA. In addition, 1 case was mosaic for DS and one case had partial trisomy [26].

**Table 1:** BBDP discovery cohort and UCI validation cohort

Characteristics	BBDP cohort	UCI cohort
Total (*n*)	251	148
Age (years), mean (SD)	85 (8.20)	78.92 (13.71)
Time (years), mean (SD)	1.77 (1.26)	3.78 (0.76)
Sex, no. (%)		
Female, *n* (%)	101 (40)	65 (43.92)
Male, *n* (%)	150 (60)	83 (56.08)
APOE ε4 carriers, no. (%)	67 (26.69)	59 (39.86)
Pathological diagnosis, no. (%)		
CAA, *n* (%)		
Mild, *n* (%)	85 (33.86)	38 (25.68)
Moderate, *n* (%)	38 (15.14)	25 (16.89)
Severe, *n* (%)	23 (9.16)	16 (10.81)
Non-CAA, *n* (%)	105 (41.83)	69 (46.62)
ADNC, *n* (%)	152 (60.56)	84 (56.76)
TDP-43, *n* (%)	64 (25.3)	11 (7.43)
LBD, *n* (%)	80 (31.6)	16 (10.81)
Trisomy 21, *n* (%)	N/A	18 (12.16)

### Differentially regulated plasma proteins in cases with CAA in BBDP cohort

**Fig. 1:**
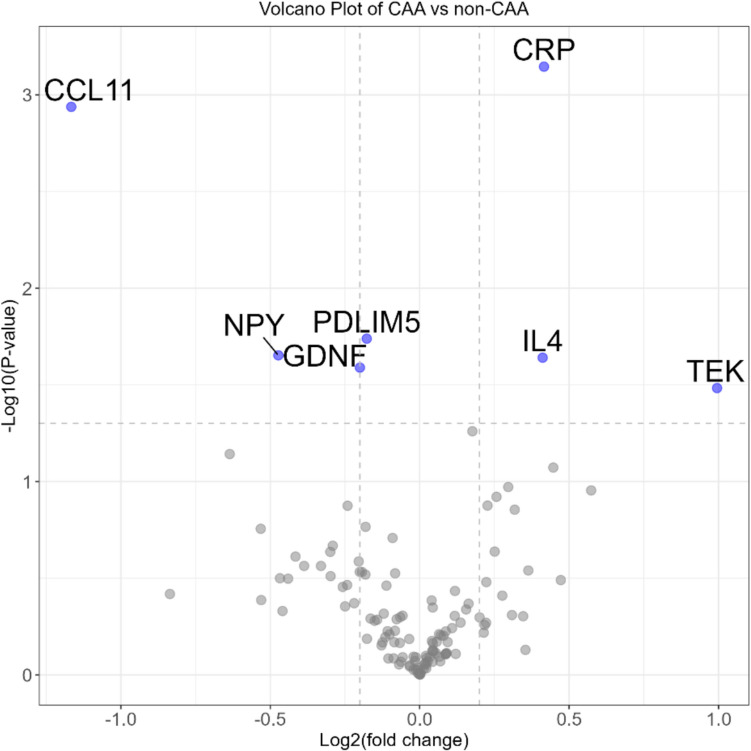
Volcano plots showing protein alterations in plasma between neuropathologically determined CAA cases and non-CAA cases. Binary logistic regression model with neuropathologically confirmed CAA-positive cases as the positive outcome evaluated the differentially regulated proteins in plasma. Blue dots indicate proteins nominally significant (*p* < 0.05 but *p*_adj_ > 0.05) which could not pass multiple testing correction. The horizontal dashed line indicates the unadjusted *P* value threshold of 0.05 while the log2fold change cut-off is set at 0.2 indicated by vertical dashed line. The *x*-axis has Log2(fold change), and the *y*-axis represents the − log10(*P *value)

We used logistic regression (adjusting for age, sex, *APOE* ε4 carriership and Alzheimer’s status) to identify the differentially regulated proteins in CAA compared to non-CAA. We identified three upregulated proteins in CAA (CRP, log_2_FC = 0.42, *P* value = 0.001; IL4, log_2_FC = 0.41, *P* value = 0.023; TEK, log_2_FC = 0.99, *P* value = 0.033) and four downregulated proteins in CAA as compared to non-CAA group (CCL11, log_2_FC = − 1.17, *P* value = 0.001; GDNF, log_2_FC = − 0.20, *P* value = 0.026; NPY, log_2_FC = − 0.47, *P* value = 0.022; PDLIM5, log_2_FC = − 0.18, *P* value = 0.018) (Fig. 1).

### Discriminatory accuracy of plasma biomarkers for CAA pathology in BBDP cohort

**Fig. 2:**
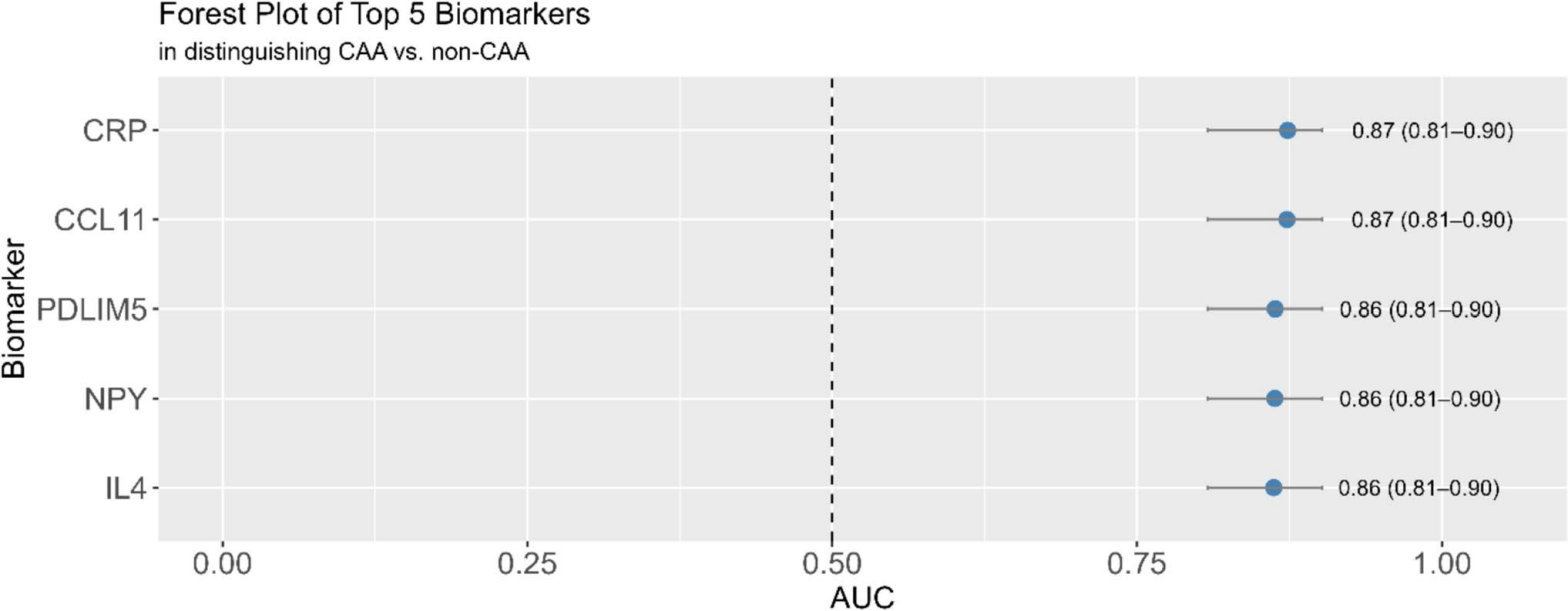
Forest plot showing the discriminatory performance of the top five protein biomarkers differentiating CAA from non-CAA. The AUC contribution of the individual biomarkers is denoted in predicting CAA while adjusting for has age, sex, APOE ε4 status and AD status. The *x* axis represents the area under the receiver operating characteristic curve (AUC). Each point represents the AUC value for a biomarker, with horizontal lines showing the 95% confidence intervals. Biomarkers are listed on the *y* axis

**Fig. 3:**
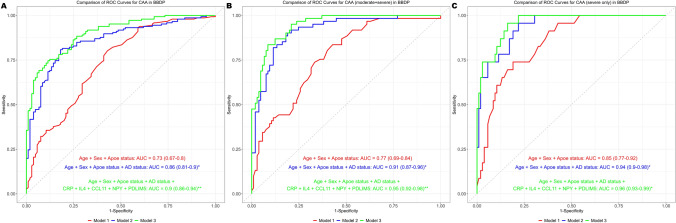
Receiver operating characteristics (ROC) curves for selected targets in detecting CAA positivity in BBDP cohort. Logistic regression and ROC curve analysis evaluated area under the curve (AUC) in predicting neuropathologically confirmed CAA-positive cases: all CAA cases (**A**), moderate and severe CAA cases (**B**), and only severe CAA cases (**C**). Basic model (Model 1) is adjusted for age, sex, APOE ε4 status while Base model (Model 2) has AD status added to Model 1. Augmented model (Model 3) has the five biomarkers added to Model 2. For each ROC curve, the AUC is reported alongside the 95% CI. *denotes *P* value (DeLong’s test) significance (< 0.05) as compared to Model 1. **denotes *P* value (DeLong’s test) significance (< 0.05) as compared to Model 2. The y-axis indicates the sensitivity, and the *x* axis indicates the specificity for each model

We calculated AICc value for all 126 proteins (Supplementary Table 1) adjusting for demographic covariates. All the biomarkers individually had an AICc value ranging from 242.28 to 252.7. We then selected the top 5 biomarkers with the lowest AICc and calculated their individual contribution to AUC for predicting CAA (Fig. 2). Even though, TEK and GDNF showed up on the volcano plots as nominally significant proteins differentially regulated in CAA versus non-CAA, their individual AICc value was higher than the selected top 5 biomarkers, and their inclusion did not add any advantage to the augmented/final model in our ROC-AUC analysis. We next investigated how the selected five plasma biomarkers combined together discriminated between subjects with CAA pathology from those with non-CAA pathology, via a receiver operating characteristic (ROC) curve analysis (Fig. 3). Our basic model (Model 1) consisted of age, sex and *APOE* ε4 carriership; Base model (Model 2) was adjusted for age, sex and APOE ε4 carriership and AD status at post-mortem (Alzheimer’s neuropathology composite); and in an Augmented model (Model 3), we added the five selected proteins with the lowest AICc in addition to the components of Model 2. In Fig. 3A, we compared all the CAA cases (mild, moderate and severe) with the non-CAA via ROC-AUC analysis. We found that Model 3 had the highest AUC (0.90, 95% CI 0.87–0.94) to discriminate between CAA and non-CAA at post-mortem in BBDP. This was found to be significantly greater than both Model 1 (0.74, 95% CI 0.68–0.8; DeLong, *P* value < 0.001) and Model 2 (0.85, 95% CI 0.81–0.90; DeLong, *P* value < 0.001). In Fig. 3B, we compared only moderate and severe CAA cases with the non-CAA via ROC-AUC analysis. We found that Model 3 had the highest AUC (0.95, 95% CI 0.92–0.98) to discriminate between CAA and non-CAA at post-mortem in BBDP. This was found to be significantly greater than both Model 1 (0.77, 95% CI 0.69–0.84; DeLong, *P* value < 0.001) and Model 2 (0.91, 95% CI 0.87–0.96; DeLong, *P* value < 0.05). In Fig. 3C, we compared only severe CAA cases with the non-CAA via ROC-AUC analysis. We found that Model 3 had the highest AUC (0.96, 95% CI 0.93–0.99) to discriminate between CAA and non-CAA at post-mortem in BBDP. This was found to be significantly greater than both Model 1 (0.85, 95% CI 0.77–0.92; DeLong, *P* value < 0.001) but not from Model 2 (0.94, 95% CI 0.9–0.98; DeLong, *P* value = 0.131).

It is well noted that we are using a neuropathological score for Alzheimer’s disease status (AD status) in our ROC-AUC model which would not be a practical solution if we want to utilize our panel of biomarkers for *in vivo* tracking of CAA. Blood-based biomarkers such as pTau217 tracks amyloid pathology very well and has been shown to have improved diagnostic accuracy for discriminating AD from other neurodegenerative diseases [2]. Utilizing pTau217 data available from Roche (eCobas601) platform for a subset of participants (*N* = 135) for our BBDP discovery cohort, we performed a sensitivity analysis wherein we utilized the pTau217 data as a proxy of AD status rather than the end-stage neuropathological evalution. In Supplementary Fig. 1A, we compared all the CAA cases (mild, moderate and severe) with the non-CAA via ROC-AUC analysis. We found out that Model 3 had the highest AUC (0.83, 95% CI 0.76–0.89) to discriminate between CAA and non-CAA at post-mortem in BBDP. This was found to be significantly greater than both Model 1 (0.67, 95% CI 0.57–0.76; DeLong, *P* value  < 0.001) and Model 2 (0.74, 95% CI 0.66–0.83; DeLong, *P* value  < 0.05). In Supplementary Fig. 1B, we compared only moderate & severe CAA cases with the non-CAA via ROC-AUC analysis. We found that Model 3 had the highest AUC (0.84, 95% CI 0.76–0.93) to discriminate between CAA and non-CAA at post-mortem in BBDP. This was found to be significantly greater than only Model 1 (0.72, 95% CI 0.61–0.83; DeLong, *P* value  < 0.05) but not from Model 2 (0.8, 95% CI 0.7–0.89; DeLong, *P* value  = 0.19). Interestingly, Models 2 and 1 were not significantly different from each other (DeLong, *P* value  = 0.09). In Supplementary Fig. 1C, we compared only severe CAA cases with the non-CAA via ROC-AUC analysis. We found that Model 3 had the highest AUC (0.95, 95% CI 0.91–1.00) to discriminate between CAA and non-CAA at post-mortem in BBDP. This was found to be significantly greater than Model 1 (0.83, 95% CI 0.69–0.97; DeLong, *P* value  < 0.05) but not from Model 2 (0.91, 95% CI 0.82–1.00; DeLong, *P* value  = 0.24). Here again, Models 2 and 1 were not significantly different from each other (DeLong, *P* value = 0.21).

**Fig. 4:**
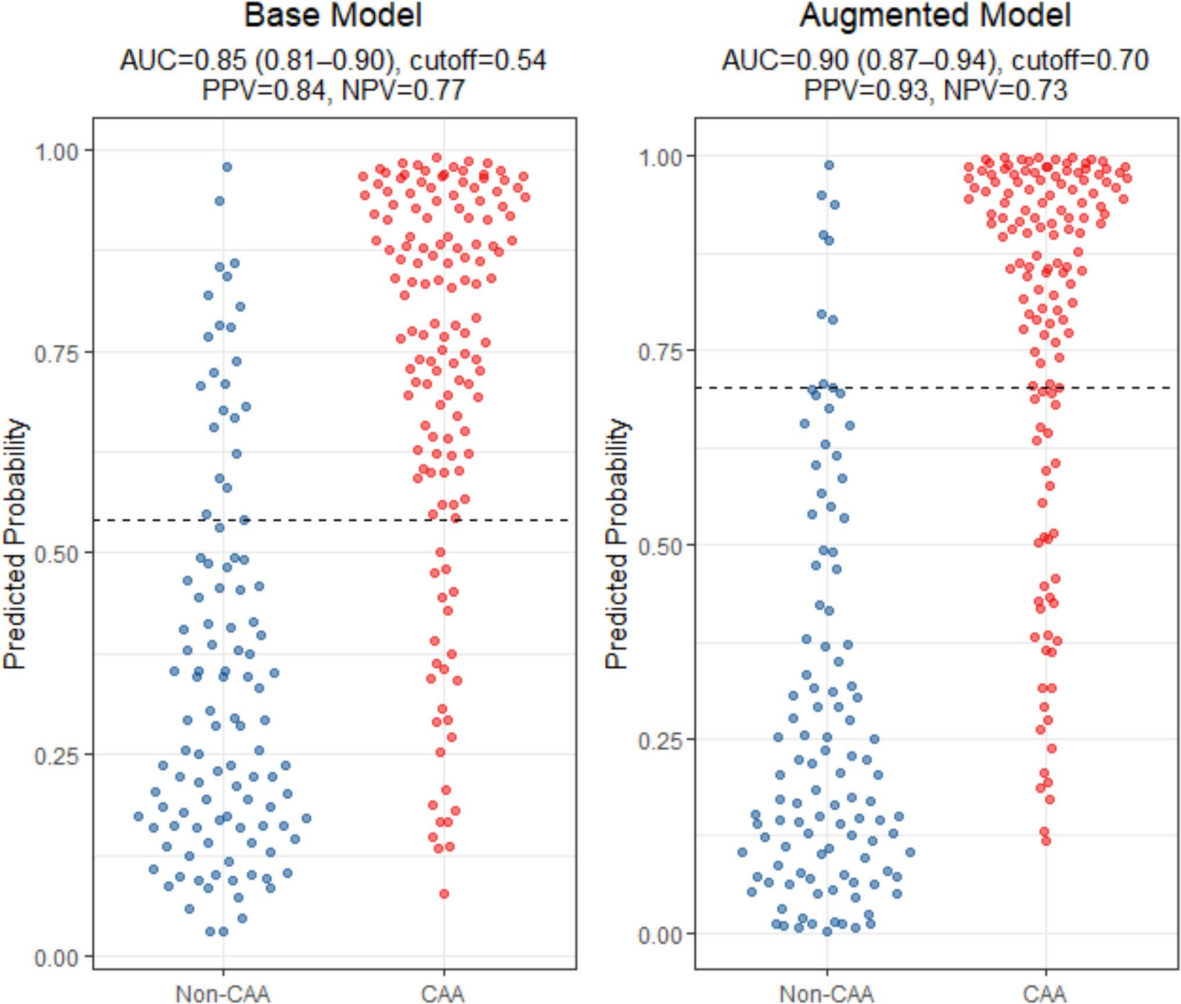
Beeswarm plots illustrating predicted probabilities from classification models stratified by disease status in BBDP cohort. Distribution of predicted probabilities of CAA positivity based on a logistic regression model is plotted. The predicted probabilities are displayed for the Base model (left) wherein the model has age, sex, AD status and APOE ε4 status as predictors. The predicted probabilities are displayed for the Augmented model (right) wherein the model includes 5 biomarkers (CRP, CCL11, NPY, PDLIM5, and IL4) along with age, sex, AD status and APOE ε4 status as predictors. The AUC, PPV, and NPV values for the respective models are denoted. Blue dots correspond to individuals who are CAA negative and red dots correspond to individuals who are CAA positive. The *y* axis indicates the predicted probability of disease for each individual. Horizontal dotted lines represent Youden's optimal cut-off threshold for classification

We also computed optimal probability cutoffs (Youden’s *J* index), along with NPV and PPV while analyzing the full dataset (all CAA cases) and visualized this with beeswarm plots (Fig. 4) wherein predicted probabilities were plotted against CAA status. For the Base model (Model 2), the Youden’s cut-off was set at 0.54 with PPV = 0.84 and NPV = 0.77. For the Augmented model (Model 3), the Youden’s cut-off was set at 0.70 with PPV = 0.93 and NPV = 0.73.

### Discriminatory accuracy of the BBDP associated biomarkers for CAA in the UCI validation cohort

**Fig. 5:**
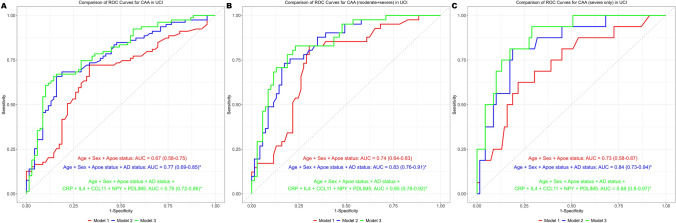
Receiver operating characteristics (ROC) curves for selected targets in detecting CAA positivity in UCI cohort. Logistic regression and ROC Curve analysis evaluated area under the curve (AUC) in predicting neuropathologically confirmed CAA-positive cases: all CAA cases (**A**), moderate and severe CAA cases (**B**), and only severe CAA cases (**C**). Model 1 is adjusted for age, sex, APOE ε4 status while model 2 has AD status added to Model 1. Model 3 has the five biomarkers (derived from BBDP cohort) added to Model 2. For each ROC curve, the area under the curve is reported alongside the 95% CI. *denotes *P* value (DeLong’s test) significance (< 0.05) as compared to Model 1. The *y* axis indicates the sensitivity, and the *x* axis indicates the specificity for each model

We next aimed to validate the accuracy of the five discovered biomarkers in discriminating between CAA and non- CAA cases in an independent validation cohort from UCI. First, we performed ROC curve analysis and compared the AUCs for each Model that was previously derived in BBDP. The analysis pipeline was similar to what was done in BBDP. First, we included all the CAA cases (mild, moderate, and severe) and performed ROC curve analysis (Fig. 5A). Here, the AUC for Model 1 was 0.67 (95% CI 0.58–0.75); Model 2 was 0.77 (95% CI 0.69–0.85); while the augmented/final model had an AUC of 0.79 (95% CI 0.72–0.86). DeLong tests between the AUCs for respective models showed that Models 2 and 3 were significantly different from the Model 1 (*P* value = 0.002 and 0.001, respectively). However, Models 2 and 3 were not significantly different. Next, we compared only moderate and severe CAA cases with the non-CAA via ROC-AUC analysis. The calculated AUCs for each model was comparable to BBDP; Model 1 was 0.74 (95% CI 0.64–0.83); Model 2 was 0.83 (95% CI 0.76–0.91); while the augmented/final model had an AUC of 0.85 (95% CI 0.78–0.92) (Fig. 5B). DeLong tests between the AUCs for respective models showed that Models 2 and 3 were significantly different from the Model 1 (*P* value = 0.008 and 0.005, respectively). However, Models 2 and 3 were not significantly different in the validation cohort. Finally, we restricted the UCI cohort to “severe” CAA cases and performed a ROC curve analysis again (Fig. 5C). We observed an increase in the AUC of the augmented/final model to 0.88 (95% CI 0.8–0.97) compared to a Model 2 AUC of 0.84 (95% CI 0.73–0.94) and a Model1 AUC of 0.73 (95% CI 0.58–0.87); but the difference between Models 2 and 3 was still not significant. It is noteworthy that 18 out of the 148 participants presented with DS (trisomy of 21st chromosome) which increases the risk of CAA due to an extra copy of APP gene [35]. However, even after exclusion of those participants, the AUC-ROC remained still the same.

As mentioned earlier, we aim to utilize the panel of biomarkers for *in vivo* diagnosis of CAA. Therefore, we did a sensitivity analysis similar to what was done in the BBDP discovery cohort utilizing the Roche (eCobas601) pTau217 data available for the participants (*N* = 148) of UCI validation cohort as a proxy of AD status in the ROC-AUC analysis. In Supplementary Fig. 2A, we compared all the CAA cases (mild, moderate and severe) with the non-CAA via ROC-AUC analysis. We found out that Model 3 had the highest AUC (0.73, 95% CI 0.65–0.81) to discriminate between CAA and non-CAA at post-mortem in UCI. This was found to be significantly greater than only Model 1 (0.67, 95% CI 0.58–0.75; DeLong, *P* value < 0.05) but not from Model 2 (0.72, 95% CI 0.64–0.80; DeLong, *P* value = 0.47). Please note that Models 2 and 1 were not significantly different from each other (DeLong, *P* value = 0.09). In Supplementary Fig. 2B, we compared only moderate and severe CAA cases with the non-CAA via ROC-AUC analysis. We found that Model 3 had the highest AUC (0.83, 95% CI 0.75–0.91) to discriminate between CAA and non-CAA at post-mortem in UCI. This was found to be significantly greater than only Model 1 (0.74, 95% CI 0.64–0.83; DeLong, *P* value < 0.01) but not from Model 2 (0.79, 95% CI 0.71–0.87; DeLong, *P* value = 0.08). Interestingly, Models 2 and 1 were not significantly different from each other (DeLong, *P* value = 0.09). In Supplementary Fig. 2C, we compared only severe CAA cases with the non-CAA via ROC-AUC analysis. We found that Model 3 had slightly better AUC (0.84, 95% CI 0.73–0.95) to discriminate between CAA and non-CAA at post-mortem in UCI. This was found to be significantly greater than Model 1 (0.73, 95% CI 0.58–0.87; DeLong, *P* value < 0.05) but not from Model 2 (0.84, 95% CI 0.74–0.94; DeLong, *P* value = 0.97). Here, Model 2 was also significantly different than Model 1 (DeLong, *P* value < 0.05).

## Discussion

We have identified a panel of five plasma biomarkers (CRP, IL4, CCL11, PDLIM5, and NPY) that could discriminate neuropathologically confirmed CAA from non-CAA cases, which was significantly greater than basic demographics, including APOE ε4 status and AD neuropathologic change. In the present study, we sought to utilize new proteomics techniques, and a *post-mortem* standard of truth, in pursuit of in vivo biomarkers for CAA that can aid patient management in the context of AD therapeutics and clinical trials. We utilized two *post-mortem* cohorts, with antemortem plasma collected within 5 years of death, for which the age, sex, APOE ε4 carriership and CAA status was available. To our knowledge, this is the first study on a neuropathologically confirmed cohort of CAA wherein plasma biomarkers for predicting CAA pathology have been identified and validated by employing the next-generation Alamar NULISA Platform with attomolar sensitivity.

Cerebral amyloid angiopathy is a common vascular disease wherein amyloid-beta deposition occurs inside the walls of small blood vessels of the brain [77]. CAA is frequently found in those with AD pathology [11, 24, 59]. Two amyloid lowering therapies, Lecanemab (Leqembi) and Donanemab (Kisunla), have demonstrated clinical benefit and entered practice [34, 54], but are currently approached with caution due to a potentially serious class-related side effect: ARIA [15, 56, 79]. The presence of CAA, along with *APOE* ε4 [21, 36], is seen as a pre-existing risk for ARIA. It is therefore crucial to identify the presence of CAA and account for it in the clinical evaluation before prescribing anti-amyloid therapy. Further, if secondary prevention of AD becomes possible with amyloid-targeting therapies, then it would be very beneficial to add a potential CAA blood-biomarker panel to the biomarker panel of AD that would likely be drawn in primary care too as a first step to identify potential candidates [49].

The definitive diagnosis of CAA is challenging, and can be done by post-mortem autopsy only, while a “probable” or “possible” diagnosis can be done in vivo [32]. Currently available methods for CAA diagnosis in vivo heavily rely on imaging techniques particularly magnetic resonance imaging (MRI). The MRI-based CAA diagnosis is often limited by the fact that the CAA-related structural lesions observed on MRI represent vascular-mediated brain damage, rather than CAA-laden vessels, thus being an indirect evidence of advanced disease consequences [46]. Hence, screening of early-stage CAA remains difficult. The last decade has marked an accelerated discovery of fluid biomarkers for AD diagnosis [1, 2, 57, 58, 76]. However, as yet, there is no fluid-biomarker indication for CAA. Therefore, just as blood-based biomarkers for AD pathology have advanced to the point of regualtory approval [58], there is a growing need for similarly accessible approaches to identify CAA, rather than relying solely on neuroimaging. The discovery of blood-based biomarkers would be an excellent complementary tool as they are more accessible, less invasive, cost-effective, and scalable.

The proteins identified in the multi-analyte biomarker profile were mainly associated with inflammatory (CRP, IL4, CCL11), neurodevelopmental (PDLIM5), and vascular functions (NPY) in the brain. Amyloid deposition in the blood vessel walls and impaired perivascular clearance in CAA may trigger an autoimmune response which may then lead to inflamed and weakened vessel walls. This in turn leads to inflammation and loss of blood–brain barrier [23, 33]. The extracellular matrix-related proteins also play an important role in maintenance of cerebrovascular structure [64]. Since the observed multi-analyte biomarkers are mainly involved in maintaining vascular structure and functions in the brain, it supports our hypothesis that their dysregulation may be involved in CAA. We validated this signature in an independent cohort by selecting the same five biomarkers from the discovery cohort. This validation cohort had an AUC of 88% and again increased the discriminatory accuracy for CAA significantly from the base demographics model.

The occurrence of CAA-related inflammation (CAA-ri) and ARIA has sparked interest in the role of neuroinflammation in CAA pathophysiology. Proinflammatory molecules, such as CRP and CCL11, have been linked to higher risk of cerebral microbleeds [16, 43, 47]. PDLIM5 is a cytoskeleton protein known to regulate neuronal development and differentiation in the brain and has been reported to be deregulated in CSF of autosomal dominant AD subjects [68]. Neuropeptide Y (NPY) is a 39-amino acid neurotransmitter that regulates cerebrovascular tone at the neurovascular unit [28] and has been reported to be significantly reduced in mouse model of AD [80]. Since CAA and AD pathology overlaps, NPY deregulation might be contributing to CAA pathology as well, as found in our study.

Our study is not without limitations. First, we validated the exploratory biomarker analysis for CAA in a validation cohort with a CAA positive prevalence significantly lower than the discovery and that used a different scoring system for both CAA and AD neuropathological change at post-mortem. Since these schema differ substantially, and rate of CAA-positivity, age, APOE carriership, and time interval between plasma collection and autopsy differed between cohorts (Supplementary Table 2), future studies should replicate our findings in independent datasets with more balanced CAA determination and prevalence. And these differences are reflected in the discriminatory accuracy of our multi-analyte panel in the discovery versus validation cohort. The AUC of the augmented model is significantly better than both Models 1 and 2 in the discovery cohort as assessed by DeLong’s test. However, the augmented model is not outperforming the Model 2 in the validation cohort. Despite these differences, we are encouraged that our plasma biomarker model significantly improved the assignment of CAA. Second, our premise is that the CAA specific biomarkers will predict the ARIA risk in subjects undergoing anti-amyloid therapies but we were unable to directly test this hypothesis, with further studies needed to establish whether baseline levels of the proteins in this biomarker panel would be predictive of those at risk and subsequently develop ARIA. Third, we do not have the MRI imaging data (Boston criteria) for cerebral microbleeds in our cohorts, hindering our ability to compare or compliment the blood biomarker panel’s performance with the current clinical CAA standards. Fourth, our model has AD status, a neuropathologically derived covariate, which makes it impossible to generalize these results in an in vivo cohort. However, we propose that in near future, combination of AD specific blood biomarkers (pTau217 + MTBR-243) [38, 39, 51] which could track both amyloid and tau pathology has the potential to replace the Alzheimer’s disease specific neuropath component of our models.

In conclusion, we have preliminarily identified a multi-analyte plasma biomarker panel that, when combined with clinically relevant characteristics and neuropathological composite measures of amyloid plaque and tangle, predicts CAA pathology with an AUC of 90%, and demonstrated its reproducibility in an independent validation cohort. It is to be noted that in our validation cohort, Models 2 and 3 (Model 2 + 5 biomarkers) were not significantly different, but this could be attributed to the difference in the scoring scheme of the two neuropath cohorts. This is a unique study which serves as an autopsy-anchored proof-of-concept for discovering novel biomarkers of CAA. This study provides preliminary evidence toward a foundational first step for development of a plasma biomarker panel that may hold potential for better antemortem identification of CAA that, with further development and validation, may potentially inform ARIA risk-predictive models. This study could serve as hypothesis-generating for testing personalized benefit–risk estimates in AD amyloid plaque-lowering mAb therapy, and possibly mitigating or managing ARIA adverse effects. Thus, with further validation in additional cohorts, it could serve as a rapid, non-invasive, and cost-effective tool for clinical application and participant selection in AD trials.

## Supplementary Information

Below is the link to the electronic supplementary material.

Supplementary file1 (PDF 660 kb)

## Abbreviations

AD: Alzheimer’s disease
Aβ: Amyloid beta
NFT: Neurofibrillary tangles
LBD: Lewy body disease
CAA: Cerebral amyloid angiopathy
APP: Amyloid precursor protein
ARIA: Amyloid-related imaging abnormalities
SVD: Small vessel disease
cSS: Cortical superficial siderosis
PET: Positron emission tomography
NULISA: Nucleic Acid-Linked Immuno-Sandwich Assay
BBDP: Banner Brain and Body Donation Program
UCI: University of Califiornia Irvine
NPQ: NULISA Protein Quantification
ROC: Receiver operating characteristic
AUC: Area under the curve
NPY: NeuropeptideY
