# Supplementary material for: A plasma protein signature for cerebral amyloid angiopathy
Source: Acta Neuropathol. 2026 May 20;151(1):59. doi: 10.1007/s00401-026-03030-5 (PMC13190554; doi:10.1007/s00401-026-03030-5)
Supplement: Supplementary file 1 — Supplementary file1 (PDF 660 kb) [file 401_2026_3030_MOESM1_ESM.pdf]

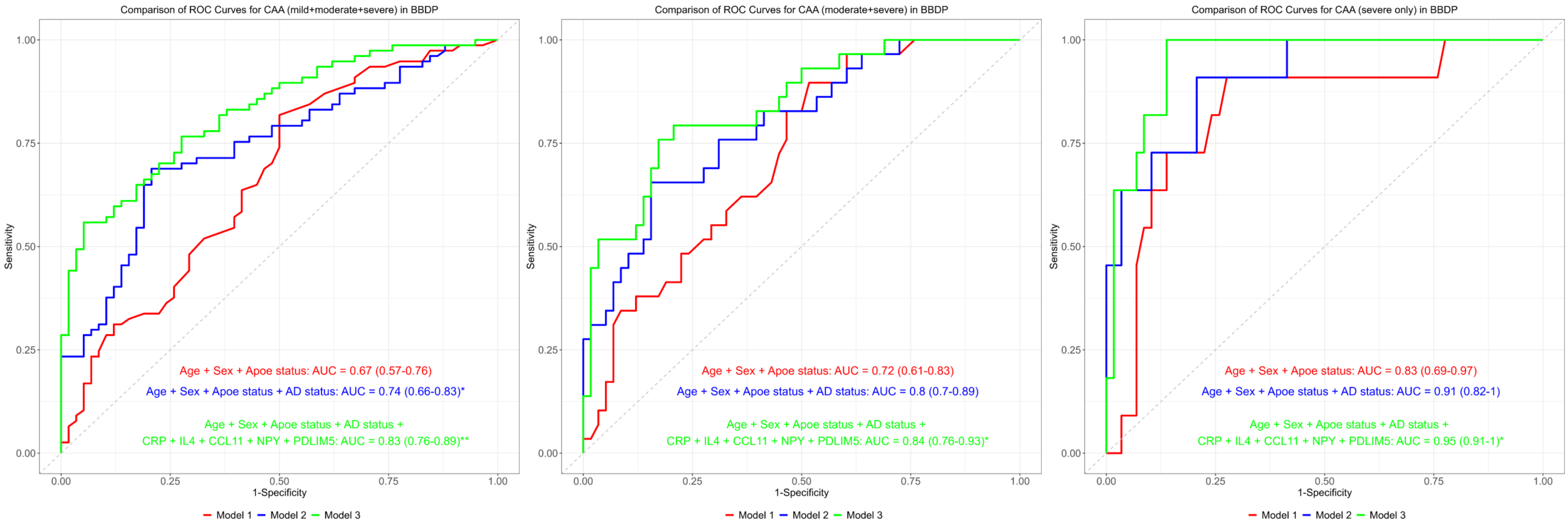

Supplementary Figure1: Receiver operating characteristics (ROC) curves for selected targets in detecting CAA positivity in BBDP cohort using pTau217 data as AD status. Logistic regression and ROC Curve analysis evaluated area under the curve (AUC) in predicting neuropathologically confirmed CAA-positive cases: all CAA cases (A), moderate and severe CAA cases (B), and only severe CAA cases (C). Basic model (Model 1) is adjusted for age, sex, APOE ε4 status while Base model (Model 2) has AD status added to Model 1. Augmented model (Model 3) has the five biomarkers added to Model 2. For each ROC curve, the AUC is reported alongside the 95%CI. *denotes p-value (DeLong's test) significance (< 0.05) as compared to Model 1. **denotes p-value (DeLong's test) significance (< 0.05) as compared to Model 2. The y-axis indicates the sensitivity, and the x-axis indicates the specificity for each model.

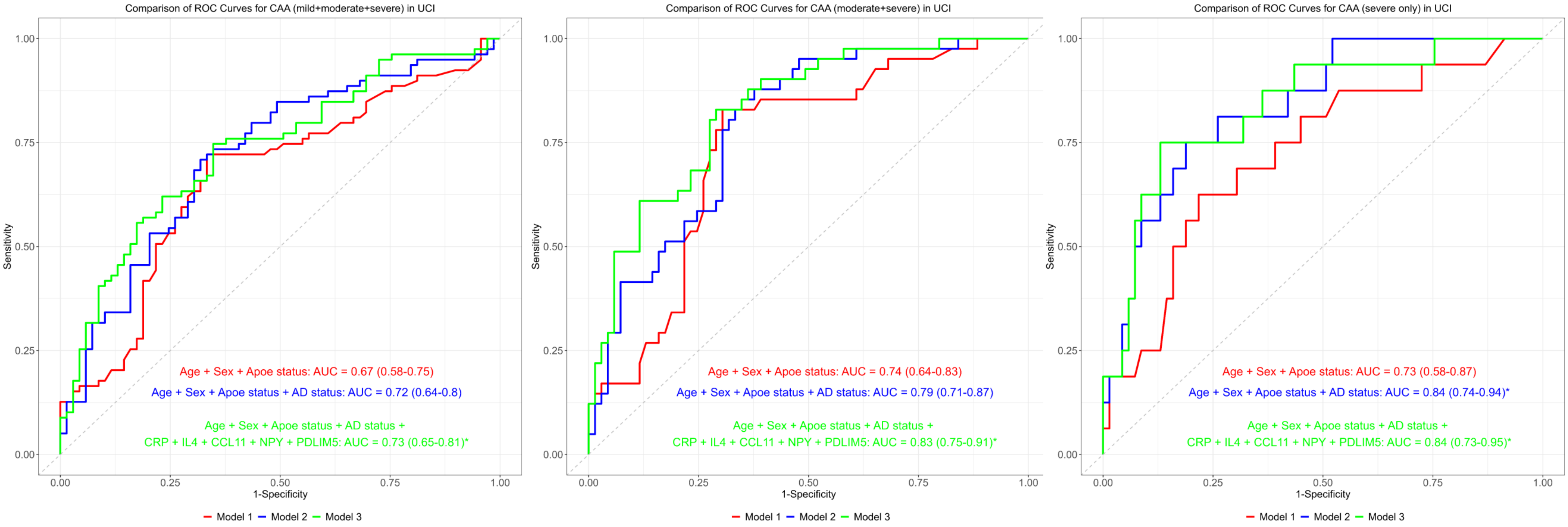

Supplementary Figure2: Receiver operating characteristics (ROC) curves for selected targets in detecting CAA positivity in UCI cohort using pTau217 data as AD status. Logistic regression and ROC Curve analysis evaluated area under the curve (AUC) in predicting neuropathologically confirmed CAA-positive cases: all CAA cases (A), moderate and severe CAA cases (B), and only severe CAA cases (C). Basic model (Model 1) is adjusted for age, sex, APOE ε4 status while Base model (Model 2) has AD status added to Model 1. Augmented model (Model 3) has the five biomarkers added to Model 2. For each ROC curve, the AUC is reported alongside the 95%CI. *denotes p-value (DeLong's test) significance (< 0.05) as compared to Model 1. The y-axis indicates the sensitivity, and the x-axis indicates the specificity for each model.

Supplementary Table 1: The Corrected Akaike's information criterion (AICc) values for all the 126 proteins from NULISA CNS panel are tabulated while adjusting for the demographic covariates (age, sex, APOE ε4 status and AD status).

Predictor	auc	aic	aicc	bic
CRP	0.862708	242.032	242.3762	263.1847
CCL11	0.864259	242.2047	242.5489	263.3574
IL4	0.855343	246.4882	246.8325	267.6409
PDLIM5	0.855602	246.6541	246.9984	267.8069
NPY	0.859736	246.7757	247.12	267.9284
TEK	0.854503	248.1012	248.4455	269.2539
GDNF	0.854374	248.4398	248.784	269.5925
SAA1	0.851596	248.8014	249.1456	269.9541
IFNG	0.854955	249.1089	249.4532	270.2616
ICAM1	0.852048	249.1517	249.496	270.3044
CSF2	0.852823	249.2003	249.5446	270.353
CXCL1	0.854632	249.6459	249.9901	270.7986
CXCL10	0.851725	249.6461	249.9903	270.7988
IL15	0.853599	250.0255	250.3697	271.1782
CCL2	0.851273	250.1878	250.5321	271.3406
IL6	0.850174	250.4155	250.7598	271.5683
IL10	0.85095	250.4479	250.7922	271.6006
FABP3	0.850627	250.4719	250.8162	271.6246
GFAP	0.849141	250.7059	251.0501	271.8586
MSLN	0.851402	250.8134	251.1577	271.9662
Aβ42	0.851725	250.9224	251.2667	272.0751
ENO2	0.850045	250.9892	251.3335	272.1419
BDNF	0.851079	251.0334	251.3777	272.1861
IL2	0.849464	251.0572	251.4015	272.2099
NEFH	0.849205	251.073	251.4172	272.2257
Aβ40	0.850691	251.1038	251.448	272.2565
IL18	0.847719	251.1638	251.5081	272.3165
AGRN	0.851337	251.1969	251.5412	272.3496
VSNL1	0.849528	251.2041	251.5484	272.3568
BACE1	0.850045	251.2077	251.552	272.3604
TNF	0.848172	251.3412	251.6854	272.4939
SLIT2	0.85095	251.3505	251.6948	272.5033
GDF15	0.849528	251.367	251.7113	272.5197
TREM1	0.851014	251.4027	251.7469	272.5554
PSEN1	0.849141	251.4076	251.7519	272.5604

CCL26	0.85011	251.4341	251.7784	272.5869
KDR	0.849722	251.475	251.8192	272.6277
NRGN	0.851402	251.4831	251.8274	272.6358
NPTX1	0.850239	251.4998	251.8441	272.6525
MME	0.848947	251.5245	251.8687	272.6772
IL7	0.849012	251.5468	251.8911	272.6995
VEGFA	0.850045	251.5495	251.8937	272.7022
SNAP25	0.849399	251.5563	251.9005	272.709
IGFBP7	0.850821	251.584	251.9283	272.7367
VCAM1	0.849981	251.6028	251.947	272.7555
IL5	0.849399	251.6052	251.9495	272.7579
TAFA5	0.851337	251.6068	251.9511	272.7596
APOE	0.850433	251.6309	251.9751	272.7836
FCN2	0.84843	251.658	252.0023	272.8107
SFTPD	0.848042	251.7712	252.1154	272.9239
CXCL8	0.849076	251.8017	252.146	272.9544
IL13	0.848882	251.8125	252.1568	272.9652
Aβ38	0.850885	251.8666	252.2109	273.0193
S100B	0.850885	251.8723	252.2165	273.025
TREM2	0.849335	251.8868	252.231	273.0395
CNTN2	0.85011	251.9071	252.2513	273.0598
CCL22	0.847073	251.9078	252.2521	273.0606
CD63	0.849722	251.9263	252.2705	273.079
HBA1	0.848882	251.9312	252.2754	273.0839
IGF1R	0.849851	251.9381	252.2823	273.0908
NPTX2	0.849141	251.9713	252.3155	273.124
PGF	0.849528	251.999	252.3433	273.1517
SNCB	0.84927	252.0031	252.3473	273.1558
CCL4	0.849141	252.0036	252.3478	273.1563
FOLR1	0.849981	252.0105	252.3548	273.1632
CCL13	0.849464	252.0117	252.356	273.1644
YWHAG	0.849141	252.0419	252.3861	273.1946
CST3	0.849205	252.0487	252.393	273.2014
CX3CL1	0.848559	252.0555	252.3997	273.2082
SQSTM1	0.848559	252.0586	252.4028	273.2113
ACHE	0.84843	252.0893	252.4335	273.242
IL9	0.848882	252.0904	252.4347	273.2432

PRDX6	0.850627	252.113	252.4573	273.2657
NGF	0.848753	252.1312	252.4754	273.2839
S100A12	0.84927	252.1335	252.4778	273.2862
IL16	0.849464	252.1409	252.4851	273.2936
CCL17	0.848689	252.1523	252.4965	273.305
pTau_217	0.848365	252.1731	252.5173	273.3258
FLT1	0.848689	252.1791	252.5233	273.3318
BASP1	0.849141	252.1906	252.5349	273.3433
IL6R	0.849076	252.1947	252.539	273.3474
UCHL1	0.849658	252.2017	252.546	273.3544
IL17A	0.848753	252.21	252.5542	273.3627
UBB	0.849464	252.2179	252.5622	273.3706
SNCA	0.849593	252.2413	252.5856	273.394
GOT1	0.849012	252.2448	252.5891	273.3975
ARSA	0.849528	252.2461	252.5903	273.3988
SFRP1	0.850304	252.2617	252.606	273.4144
NEFL	0.849658	252.263	252.6072	273.4157
GDI1	0.848042	252.2631	252.6074	273.4158
PGK1	0.847849	252.2689	252.6131	273.4216
pTau_181	0.848753	252.2692	252.6135	273.4219
IL1B	0.849528	252.2702	252.6145	273.4229
SMOC1	0.849528	252.2709	252.6151	273.4236
DDC	0.848947	252.2761	252.6204	273.4289
PARK7	0.849012	252.277	252.6213	273.4298
pTDP43_409	0.848818	252.2777	252.6219	273.4304
KLK6	0.849528	252.2838	252.6281	273.4366
IL12p70	0.850239	252.2905	252.6348	273.4432
NPTXR	0.848624	252.2919	252.6362	273.4446
ANXA5	0.848236	252.2993	252.6436	273.452
pTau_231	0.848753	252.3048	252.649	273.4575
VEGFD	0.848818	252.3058	252.6501	273.4585
HTT	0.849981	252.3066	252.6508	273.4593
REST	0.84927	252.3105	252.6547	273.4632

MDH1	0.849658	252.3237	252.6679	273.4764
FGF2	0.849722	252.3239	252.6682	273.4766
CALB2	0.848365	252.3242	252.6685	273.477
RUVBL2	0.848947	252.3244	252.6687	273.4771
TARDBP	0.848301	252.3296	252.6739	273.4824
PTN	0.848818	252.3298	252.6741	273.4826
VGF	0.848882	252.3349	252.6792	273.4876
CHIT1	0.848818	252.3371	252.6813	273.4898
TIMP3	0.848753	252.3383	252.6826	273.4911
POSTN	0.84927	252.3413	252.6856	273.494
CD40LG	0.849141	252.3415	252.6858	273.4943
PDGFRB	0.848947	252.3436	252.6878	273.4963
MAPT	0.849076	252.3464	252.6906	273.4991
SOD1	0.849141	252.3468	252.6911	273.4995
pSNCA_129	0.848753	252.3491	252.6934	273.5019
CCL3	0.849012	252.3492	252.6935	273.5019
Oligo_SNCA	0.848689	252.3506	252.6949	273.5034
CRH	0.848753	252.3518	252.696	273.5045
IL33	0.848947	252.3524	252.6967	273.5051
YWHAZ	0.849076	252.3532	252.6975	273.506
CHI3L1	0.848882	252.3536	252.6979	273.5063

Supplementary Table 2: Comparison of BBDP and UCI cohort characteristics

**Characteristic**	**BBDP** N = 251[1]	**UCI** N = 148[1]	**p-value**[2]
CAA score			0.4
CAA	146 (58%)	79 (53%)	
non CAA	105 (42%)	69 (47%)	
Age	84.99 (8.23)	78.92 (13.71)	<0.001
Sex			0.5
Male	150 (60%)	83 (56%)	
Female	101 (40%)	65 (44%)	
time (years)	1.77 (1.26)	3.78 (0.76)	<0.001
Apoe status			0.004
carrier	65 (26%)	59 (40%)	
non carrier	186 (74%)	89 (60%)	

[1]n (%); Mean (SD)

[2]Pearson's Chi-squared test; Wilcoxon rank sum test
